# Streptothrix Infections

**Published:** 1908-01-25

**Authors:** 


### Pathology in General Practice.

### STREPTOTHRIX INFECTIONS.

Not long ago it was generally believed that the effects of streptothrix infection in man were limited to a disease of the mouth and neighbouring parts known as actinomycosis, and that this disease was analogous to, and caused by the same organism as the condition known as "wooden tongue," so often met with in cattle. But it is now recognised that the term streptothrix is a generic rather than a specific name, including a number of species which may set up pathological changes in almost any part of the body, and that the particular strain of organism which infects the tongue in cattle is not, as a rule, identical with that found in human actinomycosis.

At the present time upwards of thirty species of streptothrix have been described. Some of these were obtained from the human or animal body, others from such inorganic sources as air, water, or soil. Again, a particular species can often be isolated from the interior of grains of corn and barley, and there is no doubt that this accounts for many cases of disease of the tongue in cattle. Several varieties of streptothrix—some pathogenic, others harmless—have been found in the saliva of healthy individuals, and it seems certain that, in man, the mouth is the common channel of infection, though whether this is more often conveyed by air or food is an open question. When the organism has once become established in the mouth it may develop activity in many different directions. In general the disease remains localised in the neighbourhood, appearing as a chronic indurated swelling near the mandible, in the sublingual region, or in the tongue itself. A streptothrix has also been found in an abscess at the root of a tooth, and this lends colour to the suggestion that the infection reaches the deeper tissues round the jaw by way of the socket of a diseased tooth. At other times the organisms show no sign of their presence in the mouth, but either pass downwards into the lungs, leading to a condition which resembles, according to its severity, either phthisis or bronchitis; or else they may infect any part of the alimentary canal. Streptothrix may occur, too, in association with abscess of the brain, liver, or kidney; here the path of infection probably lies through a small, unnoticed lesion of the throat or intestine. There remains a peculiar condition of the foot known as mycetoma, or madura-foot. It was at first thought that this condition could only be caused by one species, to which the name streptothrix maduræ was consequently given, but this limitation has proved to be incorrect. The infection occurs through an abrasion of the skin of the foot. It is not, at present, possible to apportion these various conditions among the members of the genus streptothrix, for a sufficient number of cases has not yet been recorded; but there is enough evidence to show that about a dozen species, which have been named and described, are possessed of marked pathogenic properties.

The clinical and histological features of the several varieties of streptothrix infection are essentially similar, and only differ slightly according to the site of infection. In the face the disease appears as a small, inflammatory nodule, slowly increasing in size, until it reaches the overlying skin or mucous membrane, which eventually give way to allow the discharge of a little yellowish fluid containing the characteristic granules. These granules are composed of a mass of coiled filaments, and are of great assistance to diagnosis. The nodule is formed by a collection of lymphocytes and endothelial cells with a few giant cells; among the cells are interspersed the mycelial threads. As the process extends there appear in the centre of the nodule small patches of necrosis, due to a fatty degeneration of the cells. If this necrosed material can be discharged, either spontaneous recovery occurs, or a sinus remains; if, on the other hand, it is pent up, these patches coalesce until a cavity containing caseous material is developed, corresponding to a tubercular abscess. When the disease is in the region of the mandible, it causes considerable erosion of the bone, or, again, it may start in the interior of the jaw and lead to expansion of the bone. In the lungs the process will, at first, be almost parallel, but the condition is often unrecognised until necrosis is far advanced, and an abscess has formed; this may point in an intercostal space, or may perforate the diaphragm and appear in the iliac region. Exceptionally the changes in the lung are more acute, and simulate purulent bronchitis or broncho-pneumonia. In the bowel a streptothrix may produce either a chronic inflammation of the appendix, or a granulamatous patch on the wall of the intestine, or an abscess in the lumbar or iliac region, derived from intestinal infection, although there may be no visible lesion of the gut. In whatever part of the body abscess-formation occurs, it is the sequel of a gradual necrosis rather than of a true suppuration, and it is doubtful whether streptothrix ever causes an acute suppurative inflammation. The changes seen in mycetoma are a conversion of the superficial structures of the sole of the foot into inflammatory granulation tissue, riddled with sinuses in all directions.

From what has been said it will be seen that, as regards their pathological effects, the streptothriceæ bear a very close likeness to the tubercle bacillus. Actinomycosis of the face is often diagnosed as tubercular, whilst it is even more difficult to give a correct opinion in many cases of infection of the lung. The histology of a streptothrix nodule follows almost exactly that of a tubercle, and the same course of events leads to the formation of an abscess in either case. Moreover, the biological characteristics of the two organisms are so closely parallel that their growth on agar and potato is scarcely to be differentiated. Finally, their staining reactions are very similar, particularly in the case of certain species of streptothrix which show the acid-fast property, so typical of the tubercle bacillus, when stained by Ziehl-Neelsen's method. It is not, therefore, surprising that an intimate relationship has been claimed for these two organisms; but, until more is known of the classification of bacteria, it will be no easy matter either to establish or to refute this claim.

